# Type I choledochal cysts in adults: natural history and implications for management

**DOI:** 10.1007/s00464-026-12935-x

**Published:** 2026-07-10

**Authors:** Sonal Walia, Mohammed Saad, James Butler, Ryan J. Ellis, Thomas K. Maatman, Trang Nguyen, C. Max Schmidt, Nicholas J. Zyromski, Eugene P. Ceppa, Attila Nakeeb, Omer Saeed, Michael G. House, Alex M. Roch

**Affiliations:** 1 Department of Surgery, Division of Surgical Oncology, Indiana University School of Medicine, 545 Barnhill Dr, EH 535, Indianapolis, IN 46202 USA; 2 Department of Pathology, Section of Gastrointestinal and Hepatobiliary Pathology, Indiana University School of Medicine, Indianapolis, IN USA; 3 Department of Surgery, Section of Surgical Oncology, Washington University in St. Louis School of Medicine, St. Louis, MO USA; 4 Division of General Surgery, Lahey Hospital and Medical Center, Burlington, MA USA

**Keywords:** Choledochal cyst, Todani type I, Adults, Cholangiocarcinoma, Bile leak, Biliary stricture

## Abstract

**Background:**

Historical pediatric data recommend surgical resection for type I choledochal cysts because of the risk of malignant progression over time (up to 60%). With the widespread use of high-resolution cross-sectional imaging, isolated dilation of the extrahepatic biliary tree has become more frequently observed. The pediatric management strategy for choledochal cysts has been extrapolated to adults, without any specific long-term data. This study aimed to characterize the natural history of choledochal cysts in adults.

**Methods:**

We have updated our previously published institutional series. From 1990 to 2023, all adult patients with a radiographic diagnosis of Todani type I choledochal cysts were identified from the endoscopic, radiologic, and surgical databases at a single academic institution. Patient demographics, clinical data, imaging studies, pathology, and management outcomes were reviewed.

**Results:**

A total of 100 patients with a median age of 41 years (range 15–78 years) and male/female ratio of 0.3 were identified. Ninety-three patients underwent surgery, including 83 (89%) with clinical symptoms with or without abnormal serologic liver function test results. Symptoms included biliary colic (62%), pancreatitis (16%), and cholangitis (11%). The median radiographic duct size was 22 mm [range 9–100]. 73% of the patients underwent Endoscopic Retrograde Cholangio-Pancreatography (ERCP) as part of their work-up. An anomalous pancreaticobiliary ductal junction (APBDJ) was identified in 83%, compared to 30% in biliary dilation from other etiology from our historical institutional cohort (*p* < 0.001). The median time from diagnosis to resection was 7 months (range 0.1–9 years). The cholangiocarcinoma rate was 6% during a median follow-up of 8 years [range 0.5–64yrs]. Five out of 6 (83%) malignant cysts were initially diagnosed during childhood, but were not resected or only bypassed. The overall morbidity after choledochal cyst resection was 50%, with Clavien-Dindo III-IV complications occurring in 40% of the patients. The clinically relevant bile leak rate was 16%, with no predictive features on the multivariate analysis. Bilioenteric anastomotic stricture and symptomatic retained cyst in the intrapancreatic portion of the bile duct requiring reoperation occurred in 24% and 5% of the patients, respectively.

**Conclusion:**

The largest Western series of type I choledochal cysts in adults confirms a lower risk of malignant transformation than previously described, occurring mostly in cysts diagnosed during childhood but not resected at that time. When diagnosed at an older age, the lower risk of cholangiocarcinoma should be weighed against the high risk of short- and long-term biliary complications associated with resection, including biliary fistula, stricture, and recurrent pancreatitis due to retained cysts within the intrapancreatic bile duct.

Choledochal cysts (CCs) are rare congenital anomalies characterized by cystic dilations in the extrahepatic and/or intrahepatic bile ducts [1]. In 1959, Alonso-Lej et al. initially classified CCs into three types based on the sites of biliary duct dilation [2]. This classification was later expanded in 1977 by Todani et al. to encompass five types [3], a nomenclature extensively adopted in present clinical practice. Type I CC, distinguished by fusiform dilation of the extrahepatic biliary tree (Fig. 1), is the most prevalent subtype in adults, noted in 68% of patients in a large multi-institutional Korean study [4].

**Fig. 1:**
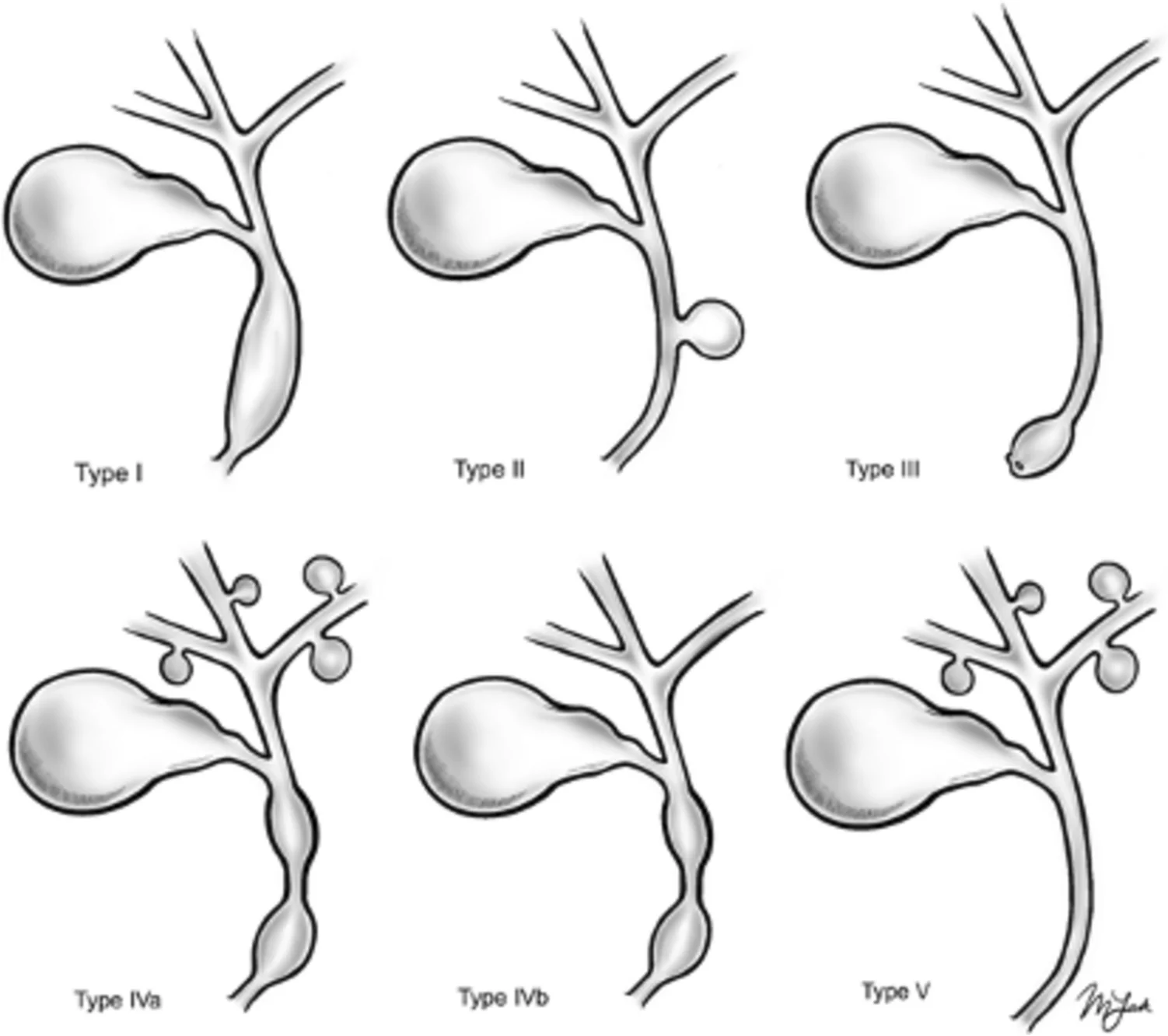
Todani classification of choledochal cysts

The precise etiology of choledochal cysts remains elusive, fueling various hypotheses. The prevailing theory, Babbitt's theory, postulates that these cysts originate from abnormal pancreaticobiliary junction (APBJ) [5]. An APBJ with a “long common channel” formed by the biliary and pancreatic ducts uniting > 10 mm proximal to the sphincter of Oddi triggers the reflux of pancreatic enzymes into the biliary tree, culminating in chronic inflammation, ductal dilation, and potential malignancy over time. Experimental canine studies have demonstrated progressive dilatation upon anastomosis of the common bile duct to the pancreatic duct, validating this theory [6].

While over 60% of patients are diagnosed during their first decade of life, with a majority within the initial year, approximately 20% persist undetected until adulthood [7]. Although CC has historically been described as a pediatric disease process, predominantly in Asia, enhancements in cross-sectional imaging have led to increased detection of CCs in adult populations over the past few decades [8].

Management recommendations for CCs primarily stem from pediatric surgery studies that advocate surgical resection due to the escalating risk of malignant transformation over time, with cancer incidences of approximately 5–10%, 20–30%, and 40–60% in the third, fourth, and fifth decades of life, respectively [9]. However, when the adult cohorts were analyzed, the risk of malignancy was significantly lower. In a pooled analysis of 78 studies, the prevalence of malignancy was 7.5% (434 cancer diagnoses in 5780 patients) [10]. Notably, the diagnosis and management of CCs may present specific challenges in adults, and the classical triad of jaundice, right upper quadrant mass, and abdominal pain is conspicuously absent in most adult cases, underscoring the importance of considering a broader spectrum of presenting features, which might feature cholangitis or pancreatitis [8, 11]. Furthermore, surgical excision may present a specific set of difficulties, particularly technical intricacies stemming from persistent inflammation and adhesions, mandating tailored treatment approaches. Additionally, two directed studies have scrutinized short- and long-term outcomes alongside the quality-of-life following CC resection, revealing a noteworthy postoperative morbidity rate of up to 34%, significantly affecting the general and mental health of individuals [12].

Observational studies of Western adult population are limited, and standardized guidelines on the optimal methods for diagnosis and care are unestablished. We hypothesized that the indications and timing of surgical resection, especially among elderly patients with multiple comorbidities, could be nuanced from the previously described guidelines. This retrospective study aimed to delineate the natural progression of CCs in adult patients, which could influence personalized therapeutic strategies for managing this unusual biliary anomaly.

## Patients and methods

### Study design and patients

A retrospective analysis was conducted on prospectively collected radiology, endoscopy, and surgery databases housing CCs information within a single adult academic institution that included cases previously published in a single institution series in 2010 [13]. The present study focused only on cases diagnosed with Todani type I CCs between 1990 and 2023. Pediatric patients aged < 15 years were excluded from the study. Other exclusion criteria included incomplete medical records, other subtypes of CCs, or absence of follow-up information. Data compilation and subsequent reporting adhered rigorously to patient confidentiality regulations set forth by the Indiana University School of Medicine Institutional Review Board.

### Definitions

Todani type I choledochal cysts were delineated according to the 1977 Todani classification [3]. All postoperative complications were graded according to the Clavien-Dindo classification system (I–V) [14]. Major complications were characterized by a severity ≥ III. Postoperative bile leaks were defined in accordance with the 2011 guidelines set forth by the international study group for liver surgery (ISGLS) [15], as a bilious intra-abdominal fluid collection on or after postoperative day three, an elevated bilirubin level in a drain, and the need for radiological intervention (i.e., interventional drainage) due to biliary collections or re-laparotomy due to biliary peritonitis. The bilirubin level in the drain was not routinely measured; however, when performed, a bile leak was defined as a bilirubin concentration at least three times higher than the serum bilirubin level measured at the same time. Clinically relevant bile leaks were defined as Grade B or C. Grade B signified bile leakage necessitating a shift in the patient's clinical management, often requiring additional diagnostic or interventional procedures, but manageable without the need for re-laparotomy. Alternatively, it could represent Grade A bile leakage persisting for over one week. Grade C indicates bile leakage requiring re-laparotomy.

### Imaging

The diagnosis of Todani type I CC was established through abdominal ultrasound (US), cross-sectional imaging studies (such as computed tomography [CT] or magnetic resonance imaging/magnetic resonance cholangiopancreatography [MRI/MRCP]), or endoscopic cholangiopancreatography (ERCP). All radiological examinations were performed by a hepatobiliary radiologist. Todani type I CC is specifically characterized by an extrahepatic bile duct exhibiting areas of cystic dilation without any discernible mechanical biliary obstruction. The presence of an anomalous pancreaticobiliary junction (ABPJ) was evaluated in all cases, with assessments undertaken most commonly by a radiologist reading the MRCP or by an advanced endoscopist conducting the ERCP.

### Pathology

**Fig. 2:**
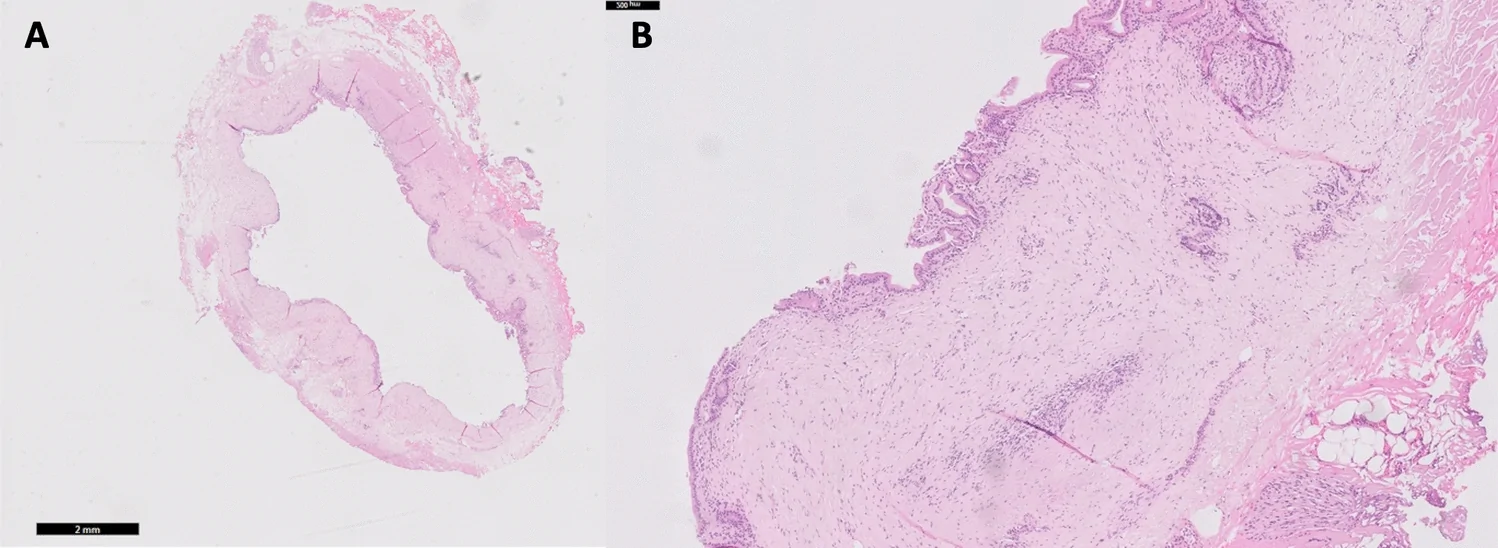
Pathological slides representative of type I choledochal cysts at low **A** and high **B** resolution. Wall of the choledochal cyst showing benign biliary epithelium, a wall with fibrosis and inflammation, and no discernable muscle layer

When resected, every specimen was meticulously examined by a gastrointestinal pathologist with expertise in hepatobiliary pathology. Histopathologically, the presence of CCs was confirmed when a biliary cyst had a wall comprising dense collagenous tissue with or without identifiable smooth muscle bundles (Fig. 2). Notably, confirmation of the diagnosis did not mandate the presence of mucosal lining. Typically, some level of inflammatory response is observed during pathological evaluation [16].

### Parameters assessed

Demographic and clinical data, including sex, age, and the presence and nature of signs/symptoms/conditions (such as abdominal/back pain, nausea/vomiting, acute pancreatitis, ascending cholangitis, cysto/hepatolithiasis, weight loss, jaundice, and portal hypertension) were ascertained from medical documentation and subjected to comparison. Various aspects were scrutinized, including the imaging technique utilized for diagnosis and endoscopic retrograde cholangiopancreatography (ERCP). Radiological and endoscopic characteristics such as bile duct diameter and the presence of an ABPJ were carefully observed and recorded. A comprehensive review of management strategies and outcomes, evaluation of the necessity for preoperative biliary stenting, instances of prior internal cyst drainage procedures, the specific type of operation performed for patients undergoing surgical resection, and the presence of short- and long-term complications classified according to the Clavien-Dindo classification system.

### Statistical analysis

Data were compiled using Microsoft Excel 2022 (Redmond, WA, USA). Continuous data were described using the mean, standard error, and range, whereas categorical variables were presented as percentages. Subgroup comparisons of continuous and categorical data were performed using Student’s t-test and Fisher’s exact test, respectively. The independent predictors of bile leak were assessed using univariate and multivariate analyses. All analyses were performed using IBM SPSS Statistics Version 27.0® (IBM Corp, Armonk, NY, IBM Corp).

## Results

### Population

During the study period, a cohort of 100 patients with Todani type I CC and the comprehensive data available for analysis were examined. The gender distribution was skewed in favor of women, with a female-to-male ratio of 3.8 (79 females and 21 males), and a median age of 41 years (range, 15–78 years). Clinically, 89% of patients experienced symptoms at the time of CC diagnosis, with biliary colic-type or right upper quadrant abdominal pain being the most prevalent (62%), followed by pancreatitis (16%), and ascending cholangitis (11%). Malignancy-suggestive symptoms were relatively infrequent, with only 6% of the population reporting weight loss.

### Radiological and endoscopic features

**Fig. 3:**
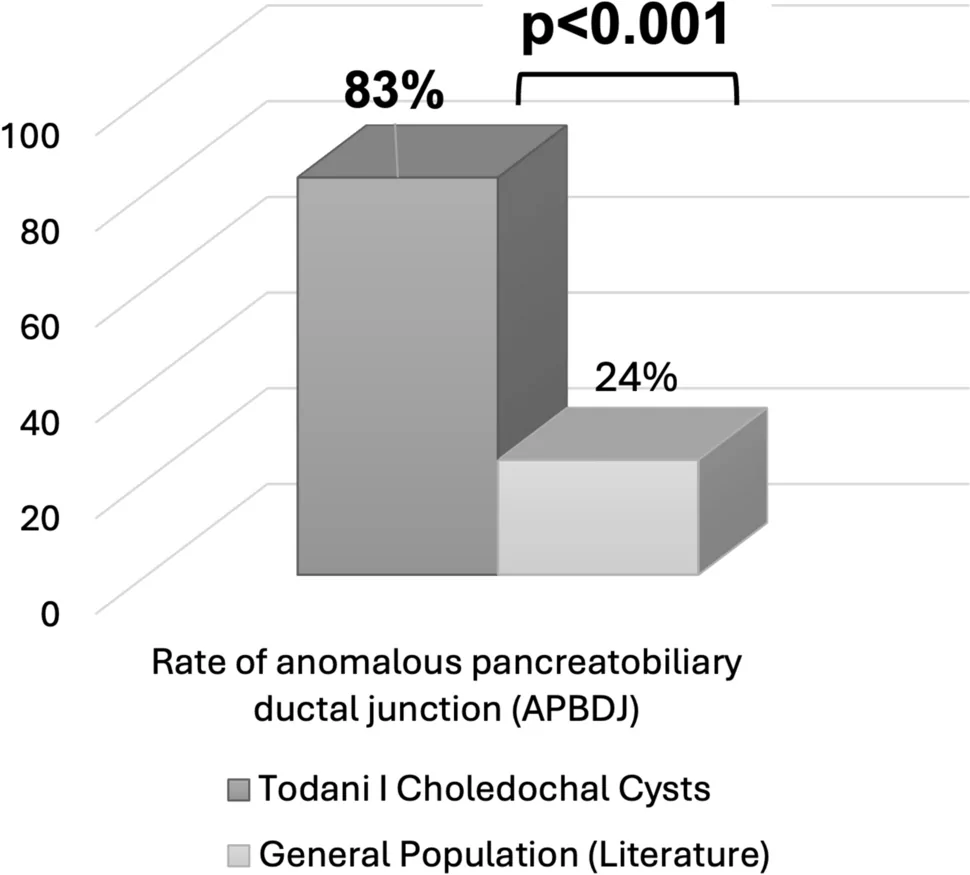
Prevalence of anomalous pancreato-biliary junction in patients with type I choledochal cysts vs. other etiologies. * Statistically significant

The majority of patients were diagnosed with type I CC through cross-sectional imaging modalities, with 54% identified using MRCP as the initial imaging modality, followed by 31% on CT scans. Abdominal ultrasound alone established the diagnosis in 10% of cases, while 5% were diagnosed through percutaneous transhepatic cholangiography. The median bile duct diameter was 22 mm, varying between 9 and 100 mm. Concordant with the Japanese guidelines for the diagnostic evaluation of biliary cysts, most patients (73%) underwent ERCP as part of their initial follow-up. Most patients (83%) exhibited evidence of APBJ on MRI/MRCP or ERCP compared to the literature rate of 24% of patients in the general population based on the literature data from Japanese nationwide survey (*N* = 1627) [17] (Fig. 3).

### Management

Among the individuals studied, 93 underwent surgical resection for type I CC, while 7% underwent serial imaging and endoscopic procedures. Symptoms prompted surgery in 89% of resected cases, with the remaining 11% undergoing surgical resection due to incidental diagnosis and the historical potential risk of malignant transformation of type I CC. The median duration between the first visit to our institution and surgical resection was 7 months, with a time range spanning from less than a month to 9 years. Of particular interest, 5 patients had a history of prior internal cystic drainage or bypass procedures during infancy without additional follow-up, adding a unique dimension to their treatment journey. The most frequently performed procedure was isolated resection of the extrahepatic bile duct (with concurrent cholecystectomy if still in situ), coupled with Roux-en-Y hepaticojejunostomy reconstruction, which was completed in 81 patients. For 9 patients, pancreatectomy was deemed necessary for comprehensive resection, and in 2 cases, partial hepatectomy was performed. Notably, only 5% of all surgeries were laparoscopically performed. Patients were followed up postoperatively for a median of 7.7 years [1 month–11.2 years].

### Malignancy

**Fig. 4:**
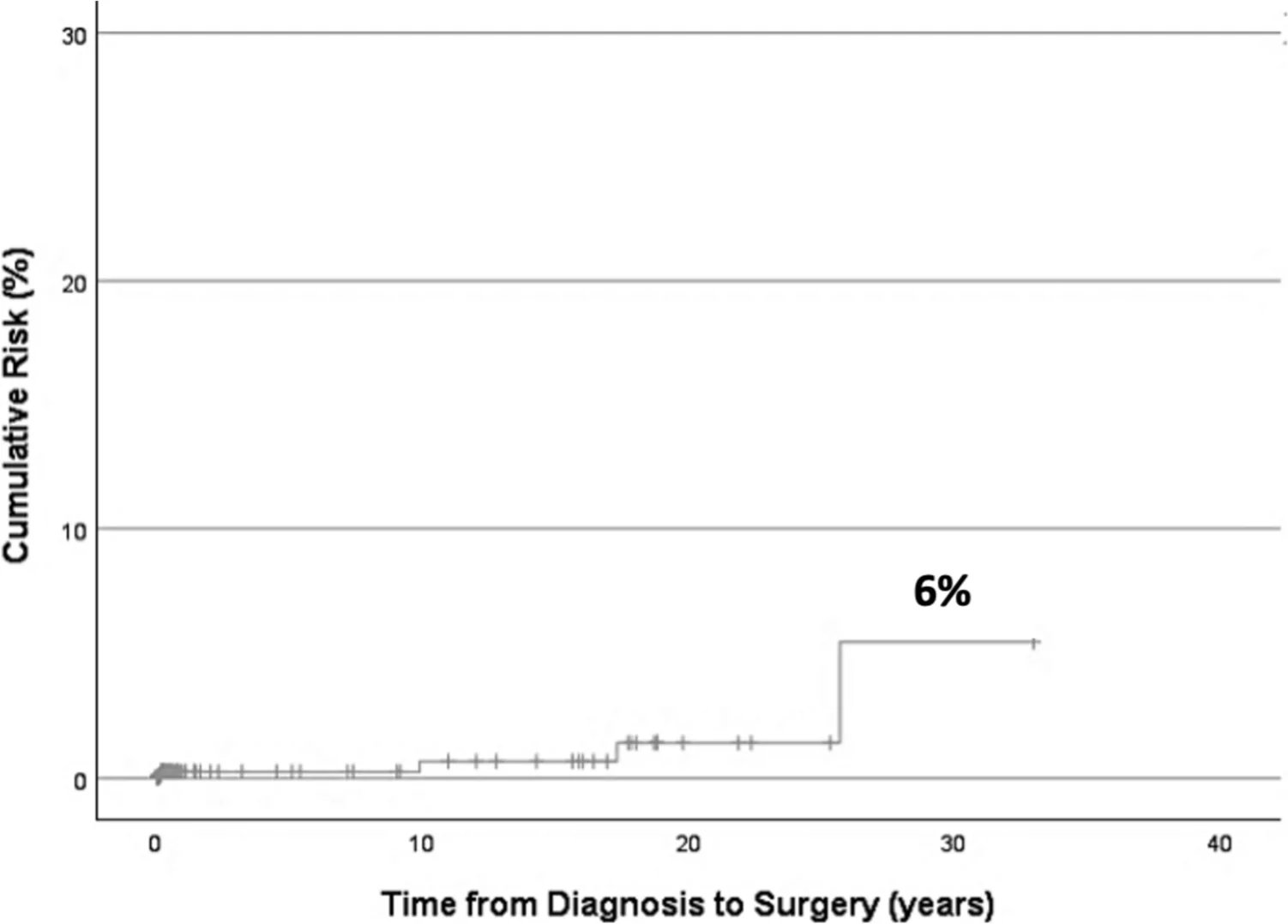
Cumulative risk of malignancy from type I choledochal cysts over time

The incidence of cholangiocarcinoma was 6% %across a median follow-up period of 7.7 years (Fig. 4). Five of six patients had invasive carcinoma identified in the final pathology following bile duct resection, and the remaining patient had a resected benign CC and later developed malignant recurrence during postoperative surveillance. Notably, in the univariate analysis, only a prolonged duration between diagnosis and surgical resection showed a statistically significant correlation with an elevated risk of malignancy (*p* < 0.001). However, it failed to show further statistical significance in multivariate analysis, and no single variable emerged as an independent predictor of malignant transformation (Table 1). All patients who developed a cholangiocarcinoma were symptomatic. Jaundice was more commonly observed in malignant CC, although the difference was not statistically significant. It is noteworthy that 5 out of 6 cases (83%) in which CCs degenerated into cholangiocarcinoma had initially been identified during infancy but were either not resected or subjected solely to drainage or bypass procedures without additional follow-up. Among these five individuals, factoring in their historical context, the median timeframe from diagnosis to progression averaged 21.5 years, ranging from 10 to 54 years. The characteristics of the six patients with malignant CC are included in Table 1.

**Table 1:** Uni- and Multivariable analysis of risk of malignancy in type I choledochal cysts

Variable	Malignant type I CC (*N* = 6)	Non-malignant type I CC (*N* = 94)	Univariate *p*-value	Multivariable *p*-value
Gender (% M)	1 (16.7%)	20 (21.3%)	0.79	-
Age at diagnosis (median ± STD, years)	38.9 ± 19.6	41 ± 19.1	0.59	-
Interval between diagnosis and surgery, when performed (median ± STD, years)	13.6 ± 20.4	4.7 ± 7.6	** < 0.001**	0.89
Symptoms (%)	6 (100%)	83 (88.3%)	0.37	–
Pancreatitis (%)	2 (33.3%)	14 (14.9%)	0.23	–
Cholangitis (%)	1 (16.7%)	10 (10.6%)	0.65	–
Jaundice (%)	2 (33.3%)	14 (14.9%)	0.23	–
Pain (%)	3 (50%)	58 (61.7%)	0.81	–
Nausea/vomiting (%)	2 (33.3%)	16 (17%)	0.31	–
Weight loss (%)	0	6 (6.4%)	0.52	–
CBD size (median ± STD, mm)	25.5 ± 33	22 ± 15.9	0.20	–
ABDJ (%)	5 (83.3%)	78 (83%)	0.98	–
Duration follow-up (median ± STD, years)	11.5 ± 24.5	7.7 ± 9.8	**0.06**	0.88

*p*-value < 0.1 in univariate analysis is highlighted in bold

### Surgical morbidity

Postoperative morbidity affected 50% of surgical patients, with 40% experiencing Clavien-Dindo III-V complications. Within this cohort, one 90-day mortality rate was recorded, accounting for 1.1% of the cases. The most prevalent complications observed were biliary in nature, affecting 35% of surgical patients (32 out of 92 patients). Recurrent acute pancreatitis from retained cysts of the intrapancreatic bile duct remnant occurred in 5% (5 of 92). Two of those 5 patients had acute pancreatitis as the presenting symptom at time of initial choledochal cyst diagnosis. Clinically significant bile leaks were noted in 16% of patients (15 of 92), while 24% (22 of 92) required interventions such as interventional radiology procedures (percutaneous biliary drain) or surgical revisions for biliary anastomotic strictures. On multivariate analysis, no variable emerged as an independent predictor of clinically relevant bile leak.

### Non-surgical patients

**Fig. 5:**
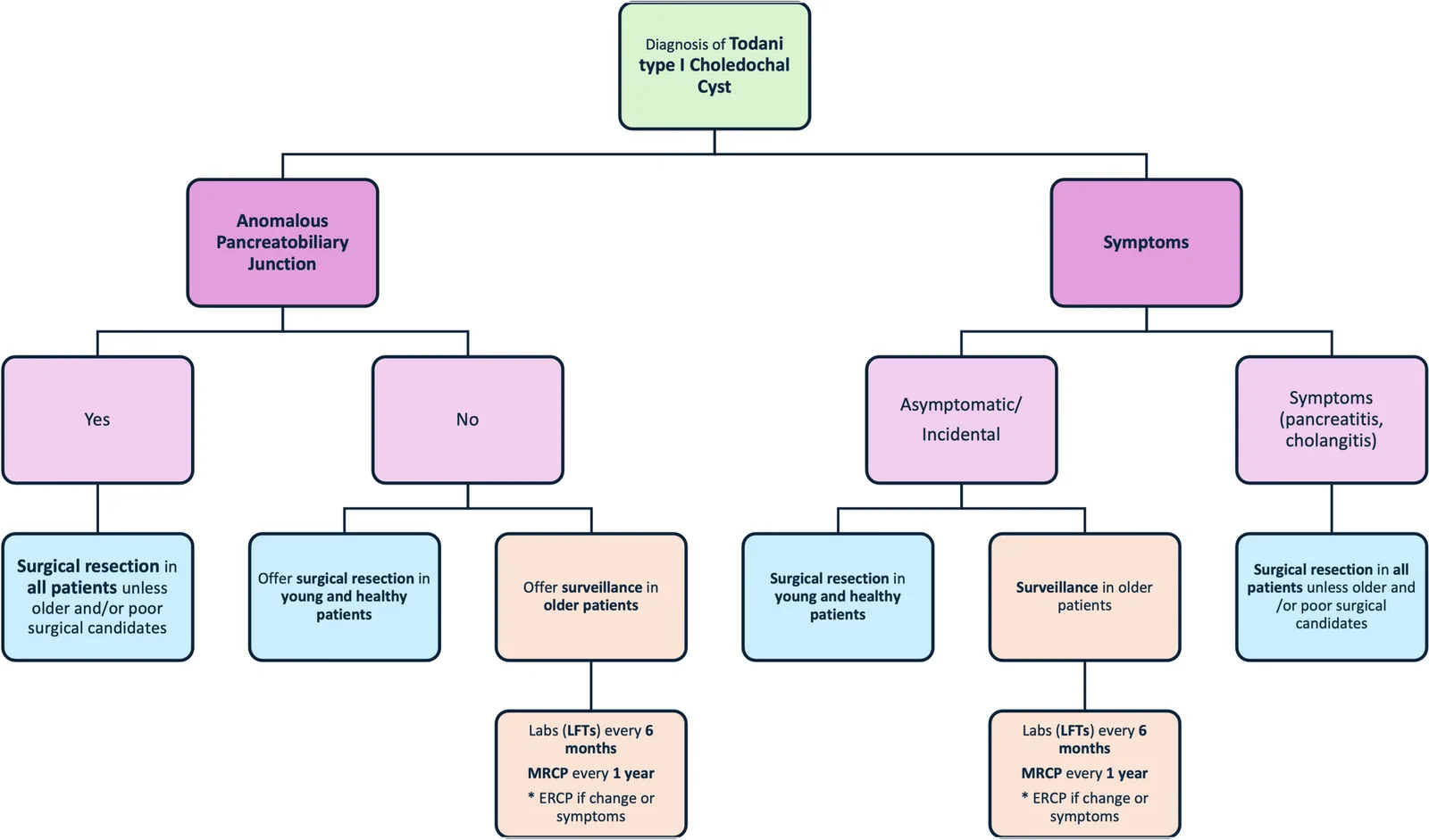
Decision algorithm for patients with Todani I choledochal cysts

In this series, 7 patients (7%) did not undergo surgical resection and were managed with serial imaging and endoscopic follow-up. The gender ratio in this subgroup was strongly in favor of women (6/1), with a mean age of 44 years old. Symptoms were present in 86% (6/7) of patients, but only 1 patient had severe symptoms at diagnosis in the form of acute pancreatitis. The median bile duct diameter was 20 mm, which was comparable to the 22 mm for the surgical subgroup (*p* = 0.54). The rate of ABPJ was 57.1% in the non-surgical group vs. 84.9% in the surgical group (*p* = 0.09). This subgroup represents a very selected cohort of patients who did not receive surgical treatment for their Todani I CC due to severe comorbidities in 3 patients (42.9%), patient’s preference in 4 cases (57.1%), including 2 completely asymptomatic patients with serial cytology from endoscopic brushing showing lack of atypical cells. None of the 7 non-surgical patients develop malignancy or required surgery on their CC during a median of 12.4 years [1.6–22.3]. A diagram summarizing the recommended management is presented in Fig. 5.

## Discussion

This study is the largest Western series offering a comprehensive analysis of type I choledochal cysts (CCs) in adults, shedding light on the natural history of these anomalies and their management implications.

Not all isolated extrahepatic biliary tree dilations in adults represent CC. In a multi-institutional study from Europe, the rationale for decision-making regarding clinical scenarios of CCs was evaluated by consultants in 30 centers [18]. Interestingly, when confronted with an incidental isolated subtle (13 mm) fusiform dilation of the extrahepatic biliary tree, external radiologists described it as a type I CC with an implicit indication for surgical resection, whereas HPB surgeons assessed it as a physiological variant (25 of 30 centers). These results suggest that the use of size alone as a diagnostic criterion to recommend surgical resection for type I CCs is not readily accepted. An important finding in our study was to validate APBJ as a key feature in distinguishing true CC from benign dilatation of the bile duct due to other causes (such as passive biliary dilation in older patients’ post-cholecystectomy).

Historical data, including the risk of malignancy up to 30%, have been empirically translated from the pediatric surgery literature into adult management [19]. With the increasing recognition of isolated dilation of the extrahepatic biliary tree in adults due to advancements in high-resolution imaging techniques, this study sought to bridge the gap in discerning the evolution of choledochal cysts in older patients and aligning management strategies with actual data tailored to this specific demographic. Interestingly, the rate of cholangiocarcinoma within this adult cohort, amounting to 6%, was significantly lower than initially postulated, but consistent with the 7% reported in the literature as “carcinoma,” both from Asia and the United States, as well as a meta-analysis of the literature [20, 21]. In our series, 83% of all cholangiocarcinomas that developed during follow-up arose from previously drained or bypassed CCs that were left in situ during infancy. This mechanism was previously reported in the literature in a French study by Ouaissi et al. in 2016, finding a statistically significant association between a history of cystenterostomy and the development of malignancy, with synchronous cancer seen in 27% of adults with a history of such drainage procedures compared to 9.7% of adults without such a history [22]. Similarly, Munoz et al. identified malignant transformation in 11% of patients with cystenterostomy after an average of 196 months (range, 12–300 months) [23]. The high rate of malignant transformation in this setting from our series may be related to a significantly longer interval from the drainage procedure to the diagnosis (up to 54 years).

The rate of malignant transformation in the overall study population must be weighed against the noteworthy morbidity profile, with up to 50% of postoperative complications and 40% of them being Clavien-Dindo III to V. These results are concordant with the 2021 National Surgical Quality Improvement Program (NSQIP) survey by Brungardt et al [24]. Biliary complications have emerged as the primary postoperative concern, affecting 35% of individuals, with 15% of patients suffering from a postoperative clinically relevant bile leak. This is similar to that reported in a nationwide Dutch cohort study published in 2020, including 123 patients with CCs (71 with Type I CC), with a bile leakage rate of 13% [25]. This incidence is significantly higher than that of bile leak after biliary bypass performed for CCs in the pediatric population or for any other pathology/etiology, which raises two main points. The relatively safe profile of biliary bypass surgery in the pediatric population (3% bile leak from a systematic review and meta-analysis published in 2021 by Hinojosa‐Gonzalez [26]) cannot be extrapolated to the adult population. Second, this increased incidence raises concerns for a different underlying process secondary to bile stasis, such as bile microbiome changes and Enterococcus overgrowth, which have been linked to an increased risk of fistula in other disease processes such as post-pancreatoduodenectomy [27].

Although there was no association with malignant potential, another notable complication described in our study was recurrent acute pancreatitis episodes stemming from the intrapancreatic bile duct remnant noted in 5% of the cases. Notably, all patients had previously undergone ERCP with sphincterotomy. This under-reported and under-defined complication certainly emphasizes the need for complete surgical resection of type I CCs. This contradicts a recent publication from China in 2018 [28], which concluded that compared to complete excision, the establishment of proper bile flow exerted a greater impact on improving long-term clinical outcomes after bile duct cyst surgery. These discordant results are related to the focus of the study, as the primary outcomes of the Chinese report were the occurrence of long-term postoperative complications (recurrent cholangitis, bile duct stones, biliary stricture, and cancer) and long-term postoperative biliary function, none of which included acute pancreatitis symptoms.

Despite representing the largest Western institutional series on Todani Type I CC, this study has several limitations. Although prospective institutional surgical, endoscopic, and interventional radiological databases were queried, and despite an extended time span, the number of patients in this study remained low compared to the Asian series. This relatively low sample size may have underestimated the true rate of malignant transformation. Similarly, the true denominator of CCs is unknown. Not all patients with CCs become symptomatic and may therefore not be diagnosed at all, contributing to an underestimation of the true prevalence. Furthermore, as described above, the imaging criteria for diagnosing type I CCs are not uniform between medical specialties or even between surgeons. Incidental extrahepatic biliary dilations may have been overlooked as benign or passive dilations, despite truly representing a type I CC.

In conclusion, this study serves as a cornerstone for expanding our understanding of type I choledochal cysts in Western adults, accentuating the critical balance between managing the risk of malignant progression and navigating the complexities of postoperative complications. In older patients for whom the risk of malignant transformation seems low, the risk of a morbid bile leak associated with a biliary bypass in this context outweighs the oncological benefit. This study offers new perspectives for management and for research. On one hand, it seems that given the high risk of biliary leak, mitigation strategies at time of surgery such as transhepatic transanastomotic drains (i.e., Heyer Shulte) should be strongly considered, especially in frail patients. On the other hand, the present study also suggests that deeper exploration of the molecular mechanisms driving the transformation of CCs into cholangiocarcinoma is needed cto identify novel therapeutic targets and optimal surveillance protocols.

## Abbreviations

ERCP: Endoscopic retrograde cholangiopancreatography
LFTs: Liver function tests
MRCP: Magnetic resonance cholangiopancreatography
